# Ofatumumab in chronic inflammatory demyelinating polyradiculoneuropathy associated with monoclonal gammopathy of undetermined significance: a case report

**DOI:** 10.3389/fimmu.2026.1633753

**Published:** 2026-04-13

**Authors:** Min Deng, Jing Xiong, Zhaohong Kong, Ying Yu, Tao Li

**Affiliations:** Department of Neurology, Renmin Hospital of Wuhan University, Wuhan, China

**Keywords:** B cell, CD20, CIDP, MGUS, ofatumumab

## Abstract

This case report describes the successful use of ofatumumab, a fully humanized anti-CD20 monoclonal antibody, in a 62 years old male with monoclonal gammopathy of undetermined significance (MGUS) associated chronic inflammatory demyelinating polyneuropathy. The patient presented with acute limb weakness, glove-like paresthesia, and respiratory involvement, alongside elevated anti-ganglioside antibodies (anti-GM4 IgM and IgG) and λ-type IgM-M proteinemia. Despite initial therapies including intravenous immunoglobulin, efgartigimod, and immunosuppressants, he experienced recurrent relapses. Treatment with ofatumumab combined with methylprednisolone resulted in complete peripheral CD19+ B cell depletion, improved nerve conduction velocities, and sustained clinical remission. The findings highlight the efficacy of ofatumumab in targeting pathogenic CD20+ B cell clones, thereby disrupting autoantibody production implicated in immune mediated peripheral nerve injury. This case underscores the potential of B cell directed therapy for refractory MGUS associated neuropathies, offering a novel approach when conventional immunotherapies fail. Further multicenter studies are warranted to establish standardized protocols and optimize long term outcomes through combination regimens targeting both B cell and plasma cell compartments.

## Introduction

Chronic inflammatory demyelinating polyradiculoneuropathy (CIDP) represents an acquired autoimmune disorder affecting the peripheral nervous system, characterized by symmetrical motor sensory deficits and distinctive neuroelectrophysiological evidence of demyelination (1). Notably, 22-30% of CIDP cases present with monoclonal gammopathy of undetermined significance (MGUS), predominantly of IgM isotype (2). Emerging evidence suggests that MGUS associated monoclonal proteins may exert neurotoxic effects through multiple mechanisms, including molecular mimicry targeting peripheral nerve components such as myelin associated glycoprotein or gangliosides, complement system activation, and functional impairment of Schwann cells (3). Moreover, clonal B cell populations in MGUS may facilitate aberrant T-cell activation through defective antigen presentation, amplifying neurological injury (4). These pathophysiological insights underscore the therapeutic potential of B cell and monoclonal protein targeted interventions in MGUS associated CIDP.

Although standard immunomodulatory therapies including intravenous immunoglobulin (IVIG) and plasma exchange (PE) remain first-line interventions, approximately 30% of CIDP patients demonstrate inadequate treatment responses, with this phenomenon being particularly pronounced in the MGUS subgroup (5). This refractory phenotype often demonstrates limited responsiveness to conventional immunosuppressants such as glucocorticoids and cyclophosphamide, with prolonged use carrying substantial risks of infectious complications (5). The persistence of monoclonal protein secretion and clonal B cell expansion appears central to therapeutic resistance, positioning B-cell depletion as a promising strategy (6).

Ofatumumab, a fully humanized anti-CD20 monoclonal antibody, exhibits enhanced B cell depletion capacity through dual mechanisms including complement dependent cytotoxicity and antibody dependent cellular phagocytosis (7). Its unique epitope binding profile enables superior targeting of B cells with low CD20 expression compared to rituximab, potentially achieving more comprehensive suppression of pathogenic autoantibody production (7). We present the first documented case of MGUS associated refractory CIDP successfully treated with ofatumumab, demonstrating significant improvements in nerve conduction parameters and motor function.

## Case presentation

A 62 years old male with poorly controlled type 2 diabetes mellitus (4 years duration) developed insidious-onset symmetrical limb weakness accompanied by glove and stocking pattern paresthesia and global areflexia in March 2024. Despite progressive symptom progression, medical evaluation was delayed until early May 2024. Neurological examination at presentation revealed proximal muscle strength grade IV and distal strength grade III, loss of tendon reflexes, and decreased sensation. Laboratory findings included elevated fasting blood glucose (6.84 mmol/L; reference: 3.9–6.1 mmol/L) and triglycerides (1.88 mmol/L; reference: 0–1.7 mmol/L). Cerebrospinal fluid analysis demonstrated albuminocytologic dissociation (protein: 2.27 g/L, normal cell count). Anti- GM4 IgM were positive, and nerve conduction studies confirmed severe demyelinating polyneuropathy. The needle electromyography at this time revealed normal motor unit potentials and no spontaneous activity. The diagnosis of CIDP was made according to the European Federation of Neurological Societies/Peripheral Nerve Society criteria (8). Serological testing returned negative for antibodies including anti-MAG, anti-ganglioside, and nodal/paranodal antibodies. Initial therapy included intravenous immunoglobulin (0.4 g/kg/day for 5 days) and antidiabetic agents (metformin 500 mg twice daily, acarbose 50 mg three times daily, dapagliflozin 10 mg once daily), resulting in partial symptom resolution.

More than a month later, symptom recurrence prompted repeat investigations. Nerve conduction studies revealed mixed demyelinating and axonal damage, with serological testing showing dual positivity for anti-GM4 IgM and IgG antibodies. Efgartigimod therapy (80 mg weekly for 3 weeks) resulted in transient clinical improvement. Mycophenolate mofetil (500 mg twice daily) was subsequently maintained as post-discharge maintenance therapy.

**Figure 1:**
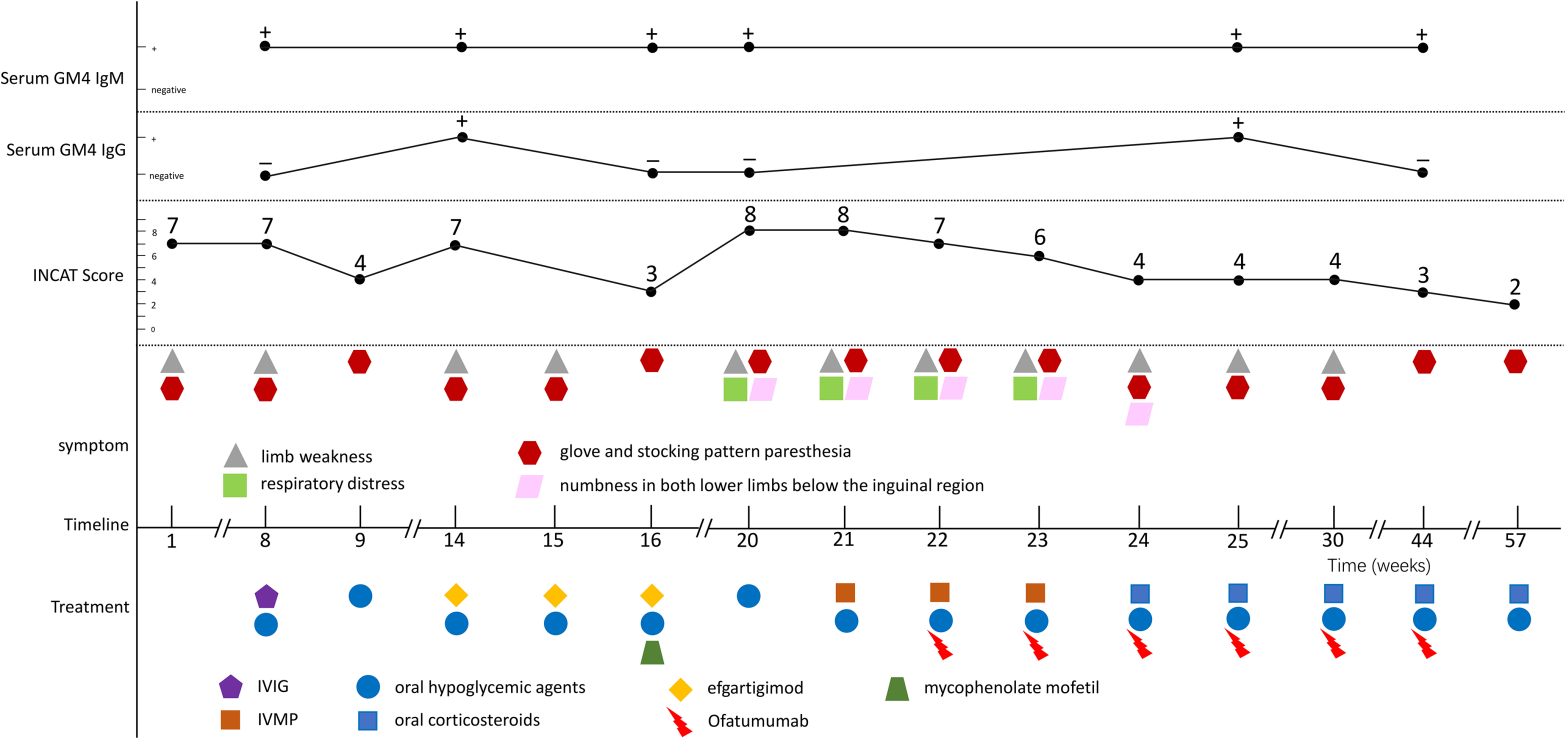
Schematic timeline of disease progression and treatment interventions in the study cohort. The illustration outlines key clinical stages, including symptom onset, administration of therapies, and the follow-up period for assessment. IVIG, Intravenous Immunoglobulin; IVMP, Intravenous Methylprednisolone.

**Figure 2:**
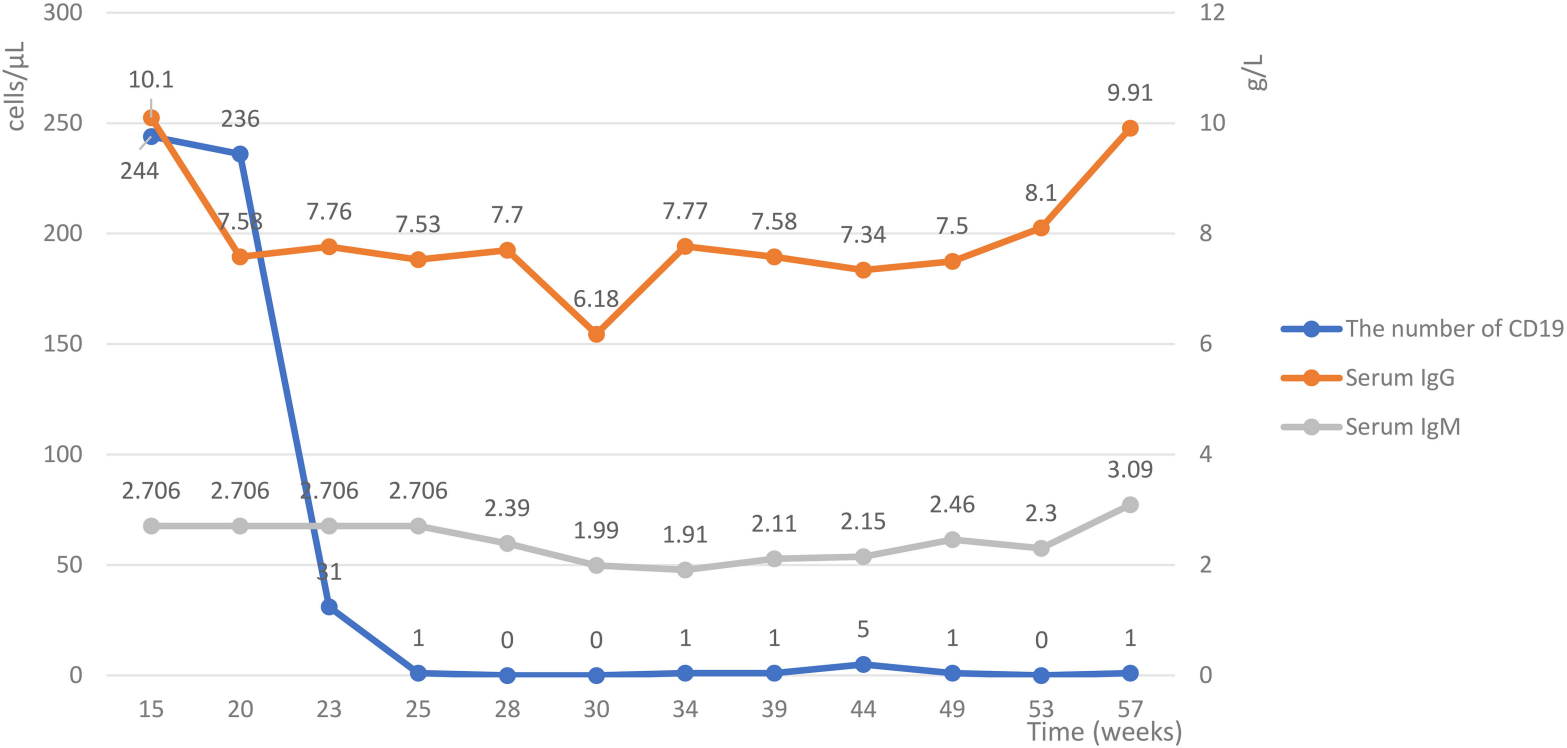
Dynamic changes in peripheral B cells and immunoglobulin levels post-treatment. Serial measurements show a rapid and near-complete depletion of circulating CD19+ B cells following therapy. In contrast, serum IgG and IgM levels exhibited fluctuation but remained detectable throughout the monitored period. IgG, Immunoglobulin G; IgM, Immunoglobulin M.

By August 2024, progressive limb weakness, respiratory distress, and numbness in both lower limbs below the inguinal region emerged. Diagnostic workup identified λ-type IgM monoclonal protein (serum M-protein: 5.01 g/L), κ-light chainuria (0.0301 g/L; reference: 0–0.02 g/L), and 0.4% monoclonal bone marrow plasma cells, confirming MGUS. Nerve studies demonstrated worsening demyelination axonal degeneration. The patient was diagnosed with MGUS associated CIDP and initiated on intravenous methylprednisolone (IVMP, 1 g/day, tapered to 30 mg maintenance) and subcutaneous ofatumumab (20 mg each time, subcutaneous injection, 6 times in total). Prior to initiating ofatumumab treatment, the patient underwent the full panel of recommended baseline investigations. All results from these investigations were within normal limits. These included a complete blood count with differential, lymphocyte subset typing, comprehensive liver and kidney function tests, serological screening for hepatitis B and HIV infection, urinalysis, and screening for latent tuberculosis infection via T-SPOT assay. Concomitant antidiabetic therapy was maintained. Post-treatment, peripheral CD19+ B cell counts dropped from 31/μL to undetectable levels. Following the initial pulse corticosteroid therapy and the first two doses of ofatumumab, the patient’s symptoms of respiratory distress gradually improved. After the subsequent four doses of ofatumumab, the symptoms had resolved entirely, leaving only distal limb numbness. Electrophysiological parameters showed improved nerve conduction velocities accompanied by enhanced functional capacity in activities of daily living. Clinical remission was sustained, supporting the efficacy of B cell depletion in disrupting autoimmune nerve injury driven by M protein and anti-ganglioside antibodies. During the course of ofatumumab treatment, no significant infectious events were observed. At the initial presentation, needle EMG revealed normal motor unit potentials (MUPs) and no spontaneous activity. In contrast, during the three subsequent follow-up examinations, needle EMG of the bilateral first dorsal interosseous, abductor pollicis brevis, tibialis anterior, gastrocnemius, and vastus medialis muscles demonstrated abnormal spontaneous activity. MUP analysis was consistent with neurogenic damage. Follow-up outcomes are detailed in **Figure 1** and **Figure 2**. The electromyography findings are detailed in **Table 1**.

**Table 1:** Electromyographic characteristics indicating motor-predominant axonal neuropathy.

Motor		First visit					1 months later			3 months later				11 months later			
Nerve	Side	DML (ms)	CMAP (mV)	CV (m/s)	TLI	DML (ms)	CMAP (mV)	CV (m/s)	TLI	DML (ms)	CMAP (mV)	CV (m/s)	TLI	DML (ms)	CMAP (mV)	CV (m/s)	TLI
Median	L	5.13	7.1	27.2	0.43	5.58	3.2	22.7	0.35	5.98	0.76	14.6	0.57	3.64	3.9	39.4	0.38
	R	7.03	2.7	30.2	0.37	5.96	1.91	23.5	0.42	7.13	0.34	19.7	0.35	4.92	1.36	40.8	0.3
Ulnar	L	4.17	5.2	39.1	0.36	4.75	4.1	26.3	0.4	5.31	1.03	22.2	0.5	3.46	3.3	39.5	0.4
	R	4.83	5	37	0.39	5.29	2.5	28.1	0.4	6.86	0.39	22.4	0.32	3.63	2.9	36.4	0.45
Tibial	L	6.77	4	—	—	9.98	1.09	—	—	11.04	0.73	—	—	5.45	4.7	—	—
	R	7.2	2.7	—	—	13	0.25	—	—	14.5	0.11	—	—	6.5	4	—	—
Peroneal	L	6.68	3.8	25.5	0.35	9.75	1.17	20.8	0.34	12.1	0.56	16.4	0.31	5.2	4.1	43	0.3
	R	6.81	3	25	0.38	7.73	0.48	15.3	0.5	9.03	0.27	13	0.51	5.6	3.8	37	0.3
Sensory						First visit			1 months later				3 months later			11 months later	
Nerve		Side				SAP (μV)	CV (m/s)		SAP (μV)	CV (m/s)			SAP (μV)	CV (m/s)		SAP (μV)	CV (m/s)
Median		L				2.9	30.5		2.6	25.7			1.94	22.5		3.2	39.8
		R				2.7	28.8		2.38	21.4			1.8	19.3		2.6	35.4
Ulnar		L				1.2	33		1	27			0.74	22		1.19	33.7
		R				1	28		0.7	23.8			0.7	21		1.64	36.2
Sural		L				2.2	30		1.85	18.4			1.45	12.7		2.4	32.5
		R				5	18.4		4.8	12.7			3.5	9.2		5.2	44.8
F-wave				First Visit				1 months later					3 months later			11 months later	
Nerve		Side		DML (ms)		F-wave Latency (ms)		DML (ms)	F-wave Latency (ms)				DML (ms)	F-wave Latency (ms)		DML (ms)	F-wave Latency (ms)
Median		L		4.3		—		5.7	—				10.5	—		3.8	41.7
		R		5.3		—		4.8	—				6.8	—		4.3	42.3
Tibial		L		7.8		—		9.2	—				—	—			—
		R		6.4		—		8.5	—				—	—			—
Ulnar		L		4.1		—		4.6	—				6	—		3	44.5
		R		4		—		5.9	—				5.2	—		3.5	46.4

Nerve conduction studies demonstrate significantly reduced amplitudes of CMAPs accompanied by decreased motor conduction velocities. SAPs were absent or markedly reduced, consistent with a sensorimotor axonal neuropathy that is more severe in motor nerves..

L, left; R, right; DML, Distal Motor Latency; CMAP, Compound Muscle Action Potential; CV, Conduction Velocity; TLI, Terminal Latency Index; SAP, Sensory Action Potential; A dash (-) indicates that data was not obtained.

## Discussion

The pathophysiological mechanism underlying CIDP associated with MGUS primarily involves B lymphocyte mediated autoimmune processes (9). Through secretion of monoclonal immunoglobulins and their cellular precursors (specifically CD20+ B cells), B lymphocytes orchestrate aberrant immune responses against neural antigens, including anti-GM4 antibodies, ultimately resulting in peripheral nerve demyelination (9, 10). Anti-CD20 monoclonal antibodies like ofatumumab exert therapeutic effects through targeted depletion of CD20+ B cell lineages. This intervention eliminates clonal plasma cell precursors responsible for M protein production, thereby diminishing autoantibody generation and interrupting downstream immune mediated neural injury (11). Emerging evidence suggests additional immunomodulatory benefits from B cell depletion, including restoration of immune homeostasis through Th1/Th2 balance modulation and enhancement of regulatory T cell functionality (12). Furthermore, this therapeutic strategy may impair B cell differentiation into antibody producing plasma cells, consequently reducing sustained release of pathogenic immunoglobulins (12). The cumulative pharmacological effects, encompassing B cell depletion, precursor cell elimination, immune regulation, and plasma cell differentiation inhibition, collectively substantiate the therapeutic rationale for ofatumumab in MGUS associated CIDP (6, 11, 12). During the treatment of this case, a temporal correlation was observed between GM4 IgG seroconversion and clinical relapses. The first relapse, characterized by limb weakness and numbness, was accompanied by seroconversion of GM4 IgG from negative to positive. After initiation of treatment with efgartigimod and mycophenolate mofetil, the GM4 IgG levels turned negative, and the patient’s symptoms gradually resolved. Similarly, during the second relapse, GM4 IgG was again detected in the serum. Subsequent sustained therapy with ofatumumab led to the disappearance of GM4 IgG and concomitant symptom alleviation.

Current treatment guidelines position CD20 targeted therapies as second line immunomodulators for CIDP, particularly in IVIG/glucocorticoid refractory cases or treatment dependent patients (13). Compared to its predecessor rituximab, ofatumumab demonstrates three distinct advantages, fully humanized structure minimizing immunogenicity risks, subcutaneous administration feasibility enhancing treatment adherence, and enhanced CD20 binding affinity enabling more sustained B-cell depletion (7). This pharmacological profile addresses key limitations of conventional therapies that fail to eradicate pathogenic B cell clones. Preclinical models suggest efficacy of ofatumumab in clearing low CD20 expressing memory B cells may translate to longer therapeutic remission (11).

Early initiation of B cell targeted therapy in treatment resistant CIDP-MGUS may confer neuroprotective benefits by preventing irreversible axonal degeneration (9). Case reports of durable remission following autologous stem cell transplantation underscore the therapeutic value of clonal B cell eradication (14). However, sustained B-cell depletion entails clinically significant risks such as hypogammaglobulinemia and opportunistic infections, necessitating systematic surveillance through serial quantitative immunoglobulin level assessments and longitudinal lymphocyte subset profiling (CD19+/CD20+ counts) to mitigate iatrogenic immunodeficiency (15).

The patient’s clinical course, characterized by symptom recurrence after initial therapies with efgartigimod and mycophenolate mofetil, followed by sustained improvement after a regimen of plasma exchange and ofatumumab, presents a complex but instructive narrative. While the temporal association of significant and sustained recovery with the initiation of ofatumumab is compelling, we must explicitly acknowledge the limitation that causality cannot be definitively established. The sequential administration of multiple immunomodulatory agents makes it challenging to attribute the clinical improvement solely to one therapy. It is plausible that the preceding plasma exchange played a critical role by rapidly reducing pathogenic antibody levels, thereby creating a more favorable immunologic environment for ofatumumab to exert its B-cell depleting effects. Therefore, the observed response is most accurately interpreted as likely resulting from the combined and sequential effect of the final therapeutic intervention. This case highlights the potential synergy of aggressive B-cell targeted therapy following plasma exchange in refractory cases, but underscores the necessity for larger, controlled studies to delineate the individual contributions of each agent.

## Conclusion

We present the first documented case of CIDP associated with MGUS achieving sustained remission with ofatumumab, demonstrating functional improvement (INCAT disability scores decreased from 8 to 2). While this success highlights the potential of generation CD20 inhibitors, several knowledge gaps persist, such as lack of standardized dosing protocols, undefined biomarkers for treatment response prediction, and unknown long term safety profile. Subsequent research efforts should focus on conducting multicenter trials that systematically incorporate serial anti-neural antibody quantification and B lymphocyte immunoprofiling, which will provide the necessary foundation for establishing data-driven clinical decision pathways.

## Data Availability

The original contributions presented in the study are included in the article/supplementary material. Further inquiries can be directed to the corresponding authors.
